# Exploring the potential of tick transcriptomes for virus screening: A data reuse approach for tick-borne virus surveillance

**DOI:** 10.1371/journal.pntd.0012907

**Published:** 2025-03-06

**Authors:** Koray Ergunay, Brian P. Bourke, Yvonne-Marie Linton

**Affiliations:** 1 Walter Reed Biosystematics Unit (WRBU), Smithsonian Institution, Museum Support Center, Suitland, Maryland, United States of America; 2 One Health Branch, Walter Reed Army Institute of Research (WRAIR), Silver Spring, Maryland, United States of America; 3 Department of Entomology, Smithsonian Institution–National Museum of Natural History (NMNH), Washington, DC, United States of America; Creighton University, UNITED STATES OF AMERICA

## Abstract

**Background:**

We set out to investigate the utility of publicly available tick transcriptomic data to identify and characterize known and recently described tick-borne viruses, using *de novo* assembly and subsequent protein database alignment and taxonomical binning.

**Methodology/principal findings:**

A total of 127 virus contigs were recovered from 35 transcriptomes, originating from cell lines (40%), colony-reared ticks (25.7%) or field-collected ticks (34.2%). Generated virus contigs encompass DNA (n = 2) and RNA (n = 13) virus families, with 3 and 28 taxonomically distinct isolates, respectively. Known human and animal pathogens comprise 32.8% of the contigs, where Beiji nairovirus (BJNV) was the most prevalent tick-borne pathogenic virus, identified in 22.8% of the transcriptomes. Other pathogens included Nuomin virus (NUMV) (2.8%), African swine fever virus (ASFV) (5.7%), African horse sickness virus 3 (AHSV-3) (2.8%) and Alongshan virus (ALSV) (2.8%).

**Conclusions:**

Previously generated transcriptome data can be leveraged for detecting tick-borne viruses, as exemplified by new descriptions of ALSV and BJNV in new geographic locations and other viruses previously detailed in screening reports. Monitoring pathogens using publicly available data might facilitate biosurveillance by directing efforts to regions of preliminary spillover and identifying targets for screening. Metadata availability is crucial for further assessments of detections.

## Author summary

Ticks can transmit many viruses with public health impact on humans and livestock. Here we explore data-reuse of publicly available tick transcriptomic or metagenome sequencing freely available in open access databases as a mechanism to passively increase xenosurveillance data on the distribution and genomic identity of tick-borne viruses. Using open-source tools for de novo assembly and taxonomic classification, we recovered 127 partial virus genomes from 35 data sets. Human and animal pathogens comprise 32.8% of the genomes, including recently described pathogens Alongshan virus (ALSV), Beiji nairovirus (BJNV) and Nuomin virus, along with African swine fever virus (ASFV) and African horse sickness virus 3 (AHSV-3). Our findings demonstrate previously generated data can be leveraged for detecting tick-borne viruses, as exemplified by records of the human pathogens ALSV and BJNV in Europe, and other viruses previously detailed in screening reports. The monitoring of pathogens using publicly available data, combined with accurate sample information, can potentially support surveillance efforts, highlighting regions of preliminary spillover and identifying targets for screening.

## Introduction

Infections caused by tick-borne viruses constitute a global zoonotic risk with substantial disease burden and public health threat and account for a major portion of the vector-borne diseases [1]. As blood-feeding vectors, ticks (*Acari*: *Ixodida*) are quintessential vessels for virus transmission, owing to their different life stages feeding on various animal hosts and potential for adaptation to diverse ecological environments. Currently, expansion of tick populations into new geographic regions with subsequent human and animal exposure is widely documented, and impacted by ongoing global climatic and environmental changes [1,2]. Increasing prevalence and geographical range have been reported for particular tick-borne viral pathogens, such as Powassan virus in North America, African swine fever virus (ASFV) in Africa and Crimean-Congo hemorrhagic fever virus (CCHFV) in Eurasia [1]. Moreover, increasing examples of novel tick-borne viruses capable of producing symptomatic human infections and severe outcomes have been documented, such as Alongshan virus (ALSV), Jingmen tick virus (JMTV), Beiji nairovirus (BJNV) and Tacheng tick virus 1 (TTV1) [3–6]. Although many of the case descriptions and screening findings originate from Asia, recent investigations demonstrate expansion of these viruses into Europe, raising questions about undiagnosed human infections occurring at the larger geographical scale [7,8]. Due to lack of commercially available nucleic acid or serology-based testing, scarce information is currently available for the distribution and public health impact of these viruses.

Timely identification of the circulating pathogens prior to disease emergence or outbreak is significantly facilitated by surveillance [9]. Monitoring the spread of viruses and identifying new pathogens in vectors and animal reservoirs constitute key steps in describing spillover, emergence and subsequent mitigation of infectious diseases caused by these agents. Vector surveillance provides an effective strategy for monitoring the introduction or circulation of emerging pathogens into susceptible populations and an early warning system for predicting outbreaks [1,9]. Direct bio- or xeno-surveillance for arthropod-borne infections are mainly based on targeted pathogen antigen or nucleic acid screening and requires biological sample collection, handling, and testing; therefore, personnel, infrastructure, and funding. Nowadays, transcriptomic or metagenome sequencing (MS) technologies have become widely available and utilized in various settings that require sequence generation, including those involving field-collected or laboratory-reared vector arthropods [10]. Enabling analysis of the nucleic acid content in the sample without prior information, MS has proved to be a robust method for virus identification, as well as describing novel virus genomes [11]. Regardless of the goals of the original investigations, MS data is frequently deposited in publicly accessible repositories, with massive amounts of sequencing data collected from various sources [12]. This exponentially growing data is likely to contain sequences originating from a wide variety of viruses, including divergent local strains and those newly described as pathogens, unknown at the time of data generation [13]. Hence, existing raw data sets in public repositories can be recycled for retrospective virus screening and might be leveraged for preliminary screening. This study was carried out to investigate the utility of publicly available tick transcriptomic data as a resource to identify and characterize known and recently described tick-borne viruses.

## Methods

### Dataset selection

High-throughput sequencing data available at the NCBI Sequence Read Archive (SRA) database [12] was initially screened during November 2023 according to the following search keywords: (“Ixodidae”[Organism]) AND (“Illumina”[Platform]) AND (“biomol rna”[Properties] AND “library layout paired”[Properties]. Search outputs and associated metadata were manually downloaded and checked for availability of information on location, sample properties and target tissues of ticks. Other runs in the bioproject associated with the selected data were further examined for additional samples. Any transcriptome lacking these metadata were omitted. Raw data available as FASTQ was used for downstream analysis. In addition, publication records linked with bioproject were examined and datasets originally generated via metatranscriptomics for virome exploration, virus screening or discovery were omitted. List of transcriptomes selected for downstream processing is provided in S1 Table.

### Virus contig generation

Demultiplexed raw Illumina data were first adaptor trimmed and quality filtered using fastp v0.23.3 (--qualified_quality_phred = 15; --unqualified_percent_limit = 40) [14,15]. The data was then de-novo assembled using MEGAHIT v1.2.9 and its “basic usage” setting for paired end libraries [16]. *De novo* assembled contigs were then aligned to the National Center for Biotechnology Information (NCBI) protein non-redundant (nr) database (ftp://ftp.ncbi.nlm.nih.gov/blast/db/FASTA/nr.gz; accessed April 5 2023) using Diamond (--long-reads; --evalue 1e-9) [17,18] and taxonomically binned using Megan v6.24.20 (--minSupport 1; --minPercentIdentity 70; --maxExpected 1.0E-9; --lcaAlgorithm longReads; --lcaCoveragePercent 51; --longReads) [19,20]. A classification table comprising sequence names and NCBI Taxonomy ID was created using the daa2info module available with the Megan v6.24.20. A list of NCBI virus Taxonomy IDs, were generated based on the International Committee for the Taxonomy of Viruses (ICTV) (https://ictv.global/taxonomy; accessed May 4 2023), using the list function in the taxonkit tool [21]. Subsequently, all virus sequence listed in classification table were extracted from assembly files using the subseq option available in the seqtk tool [22].

### Sequence handling and phylogenetic analysis

Contigs were handled using Geneious Prime (v2023.2.1) (Biomatters Ltd., Auckland, New Zealand). BLASTn and BLASTp algorithms were used in default settings used for similarity searches in the NCBI database [23]. Contigs were mapped using Minimap2 v2.24 (long assembly to reference mapping; preset option -x asm20, i.e., up to 20% divergence), and nucleotide/protein alignments were generated using CLUSTALW (full multiple alignment mode) [24, 25]. Phylogenetic relationships between virus contigs and near relatives according to ICTV were explored using maximum likelihood analysis performed in MEGA v11.0.13 [26]. The optimal model for the phylogenetic and molecular evolutionary analyses was determined using the built-in “Find Best DNA/protein-substitution model” tools. Maximum-likelihood trees based on the nucleotide sequences were constructed using the Jones-Taylor-Thornton model. The reliability of the inferred trees was evaluated by standard bootstrap analysis of 500 replicates.

## Results

**Fig 1:**
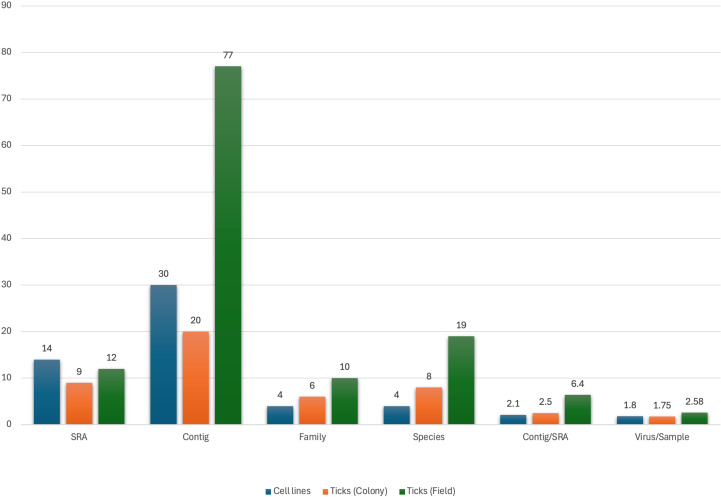
Overview and metrics of the transcriptomes, sample types and virus contigs.

**Fig 2:**
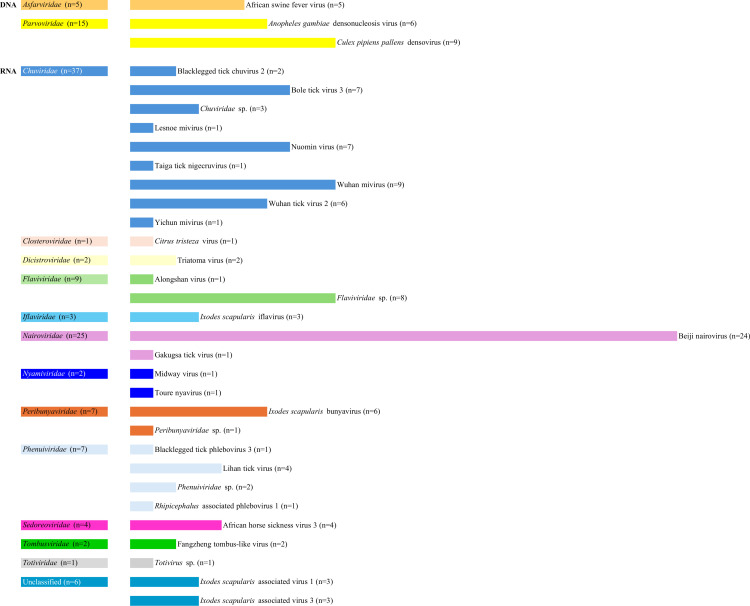
Classification and distribution of virus contigs generated in the study.

A total of 85 tick transcriptomes were processed (S1 Table), resulting in 127 virus contigs generated in 35 transcriptomes (41.2%) (Fig 1). The virus contigs originated from cell lines (30, 23.6%), colony reared ticks (20, 15.7%), and field collected ticks (77, 60.6%). The tick transcriptomes (n=21) were produced from the whole body (n=12), salivary gland (n=5), or the midgut (n=4) (Table 1). Generated virus contigs encompass DNA (n=2) and RNA (n=13) virus families with three, and 28, taxonomically distinct isolates, respectively (Fig 2). Field-collected ticks provided more virus contigs compared to other samples, with pronounced diversity by various metrics including total virus family and species counts, as well as contigs per transcriptome and viruses per sample (Fig 1) (S1 Table). In four samples originating from cell lines (C5), colony-reared ticks (L5) or field-collected ticks (F3 and F4), longer contigs of identical viruses were generated by reference mapping of the initial contigs (S2 Table).

**Table 1:** Summary of the transcriptomes and virus contigs.

		Tr. #	Tick species	Location	Host	Material	Virus	Contig #
Cell lines (n=14)	AAE1	1	*A. americanum*	KY, USA	n/a	Whole cells	*Culex pipiens pallens* densovirus	1
	DAE100	4	*D. andersoni*	OK, USA	n/a	Whole cells	*Anopheles gambiae* densonucleosis virus	3
							*Culex pipiens pallens* densovirus	3
	DVE1	6	*D. variabilis*	MN, USA	n/a	Whole cells	*Anopheles gambiae* densonucleosis virus	3
							*Culex pipiens pallens* densovirus	5
	ISE6	3	*I. scapularis*	MD, USA	n/a	Whole cells	*Ixodes scapularis* bunyavirus	6
							*Ixodes scapularis* iflavirus	3
							*Ixodes scapularis*-associated virus 1	3
							*Ixodes scapularis*-associated virus 3	3
Ticks from colonies (n=9)		8	*I. ricinus*	Switzerland	n/a	Whole body	Beiji nairovirus	6
							Lesnoe mivirus	1
							Blacklegged tick chuvirus 2	2
							Blacklegged tick phlebovirus 3	1
							*Phenuiviridae* sp.	2
		1	*R. zambeziensis*	South Africa	n/a	Salivary gland	African swine fever virus	3
							African horse sickness virus 3	4
							*Citrus tristeza* virus	1
Field-collected ticks (n=12)		4	*I. persulcatus*	China	n/a	Whole body	Beiji nairovirus	18
							Gakugsa tick virus	1
							*Chuviridae* sp.	3
							Yichun mivirus	1
							Taiga tick nigecruvirus	1
							Fangzheng tombus-like virus	2
							Nuomin virus	7
							*Totivirus* sp.	1
							*Peribunyaviridae* sp.	1
		3	*R. annulatus*	Israel	*Bos taurus*	Salivary gland	*Flaviviridae* sp.	8
							Lihan tick virus	4
							*Rhipicephalus*-associated phlebovirus 1	1
							Wuhan tick virus 2	6
							Bole tick virus 3	7
							Wuhan mivirus	9
		3	*O. erraticus*	Spain	*Sus scrofa*	Midgut	Alongshan virus	1
							Midway virus	1
							Toure nyavirus	1
		1	*O. moubata*	Malawi	*Sus scrofa*	Midgut	African swine fever virus	2
		1	*O. rostratus*	Brazil	n/a	Salivary gland	Triatoma virus	2
*Total*		35						127

Tr.: transcriptome; n/a: no information available – not relevant.

### Viruses in cell lines

Fourteen of the transcriptomes with virus contigs were originally generated in cell lines established from the embryonic tissues of laboratory reared ticks belonging to four distinct species (Table 1). A total of 30 contigs representing 6 viral taxa from 4 families were identified. Interestingly, three of the four cell lines (AAE1, DAE100 and DVE1) yielded only DNA viruses of the *Parvoviridae* family; namely, *Culex pipiens pallens* densovirus and *Anopheles gambiae* densonucleosis virus (S2 Table). In contrast, the *Ixodes scapularis* cell line (ISE6) transcriptomes yielded various tick-associated virus contigs of wider divergence, encompassing many RNA viruses of different families as well as currently unclassified isolates (S2 Table). Three of the virus contigs (*Ixodes scapularis* bunyavirus, *Ixodes scapularis* iflavirus, *Ixodes scapularis*-associated virus 3) were originally described from ISE6 cells [27], while the remainder (*Ixodes scapularis*-associated virus 1) was further detected in field-collected ticks [28].

### Viruses in colony ticks

Twenty virus contigs representing 8 taxa belonging to 6 families were generated from 8 transcriptomes from colony reared ticks, mostly comprising *Ixodes ricinus* species (Fig 1, S2 Table). Interestingly, 6 (75%) of the transcriptomes yielded tick-borne pathogen contigs including ASFV, African horse sickness virus (AHSV) 3 and BJNV. ASFV and AHSV-3 originated from the single transcriptome generated from salivary glands of *Rhipicephalus zambeziensis* from South Africa. This dataset further provided a contig of *Citrus tristeza* virus. BJNV was exclusively identified in *I. ricinus* samples, that further yielded viruses from *Chuviridae* (Blacklegged tick chuvirus 2, Lesnoe mivirus) and *Phenuiviridae* (Blacklegged tick phlebovirus 3, *Phenuiviridae* sp.) families, previously described in *I. scapularis* and *Ixodes persulcatus* ticks collected in USA and China (Tables 1 and S2) [29].

### Viruses in field ticks

Twelve transcriptomes from five tick species collected from a broad geographical distribution yielded 77 virus contigs of 19 viruses, classified in 10 families, representing a larger and more diverse cohort compared to other samples (Table 1, Fig 1). Pathogenic viruses including ALSV, ASFV, BJNV-Gakugsa tick virus and Nuomin virus (NUMV) were identified in 5 (41.6%) SRAs. The transcriptomes from *Ixodes persulcatus* and *Rhipicephalus annulatus* were observed as the main contributors to the virus diversity (Table 1). In *Rhipicephalus annulatus* transcriptomes, contigs of Bole tick virus 3, Wuhan mivirus and Wuhan tick virus 2 (*Chuviridae*), Lihan tick virus and *Rhipicephalus* associated phlebovirus 1 (*Phenuiviridae*) and unclassified *Flaviviridae* sp. were generated. These viruses have been described in *Rhipicephalus* as well as other tick species, displaying a worldwide distribution as evidenced by MS [29–31]. The *I. persulcatus* SRAs produced different viruses from related families including Taiga tick nigecruvirus, Yichun mivirus and unclassified *Chuviridae* sp. (*Chuviridae*), Fangzheng tombus-like virus (*Tombusviridae*) and *Peribunyaviridae* sp., previously documented in *Ixodes* sp. ticks, in addition to *Totivirus* sp. (*Totiviridae*), hosted by fungi in nature [29–33]. In two SRAs from *Ornithodoros erraticus* samples (F11, F12), contigs of nyaviruses (*Nyamiviridae*), mainly hosted by *Ornithodoros* ticks*,* and birds but detected in diverse invertebrates [34], were identified (S2 Table).

### Tick-borne pathogenic viruses

Virus contigs of previously documented tick-borne pathogens were identified in 11 transcriptomes (31.4%) and comprise 42 contigs (32.8%) of 6 viral taxa from 5 families (Table 2). Most prevalent pathogen was observed as BJNV (and Gakugsa tick virus, a discouraged synonym for BJNV, *Nairoviridae*) [35], identified as 25 contigs (19.5%) in 8 samples (22.8%), in colony reared *I. ricinus* (n=5) and field collected *I. persulcatus* (n=3) samples, including various developmental stages, feeding status and sex (Table 2). Both genome segments of BJNV were represented in the contigs. Alignment and maximum likelihood analyses of virus nucleoprotein and replicase sequences (encoded by S and L genome segments) in samples with contigs of sufficient sizes revealed clustering with BJNV sequences within the genus *Norwavirus* (S1 Fig). In the nucleoprotein phylogeny, while contigs from field collected *I. persulcatus* (F4, F7) grouped with BJNV-Gakudsa tick viruses (*Norwavirus beijiense* species), the contig from *I. ricinus* colony sample (L5) was placed as a separate taxon with the *Norwavirus grotenhoutense* cluster with Grotenhout and Pustyn viruses, currently classified as a separate species within *Norwavirus* genus (S1 Fig) [35]. Given the diversity in identity and coverage, some contigs identified in colony-reared ticks might indicate both virus species to be present in these transcriptomes.

**Table 2:** Tick-borne viral pathogen contigs.

Virus	Contig #	Code	Species	Location	Sample	Contig #	Genomic region
Beiji nairovirus	24	L1	*I. ricinus*	Switzerland	♂ (unfed)	1	Nucleoprotein (segment S)
		L2	*I. ricinus*	Switzerland	Nymphs (partially-fed)	1	Nucleoprotein (segment S)
		L3	*I. ricinus*	Switzerland	Nymphs (unfed)	1	Nucleoprotein (segment S)
		L4	*I. ricinus*	Switzerland	Nymphs (unfed)	1	Nucleoprotein (segment S)
		L5	*I. ricinus*	Switzerland	Nymphs (unfed)	2	Nucleoprotein (segment S)
		F4	*I. persulcatus*	China	♀ (unfed)	16	Nucleoprotein (segment S) Replicase (segment L)
		F6	*I. persulcatus*	China	♀ (engorged)	1	Nucleoprotein (segment S)
		F7	*I. persulcatus*	China	♀ (mixed)	1	Nucleoprotein (segment S)
Gakugsa tick virus	1	F4	*I. persulcatus*	China	♀ (unfed)	1	Replicase (segment L)
Nuomin virus	7	F4	*I. persulcatus*	China	♀ (unfed)	7	Replicase, Glycoprotein, Nucleoprotein, VP4
African swine fever virus	5	L8	*R. zambeziensis*	Malawi	♀+♂	3	C475L, B318L, C717R
		F9	*O. moubata*	South Africa	engorged	2	F778R, EP1242R, EP84R
African horse sickness virus 3	4	L8	*R. zambeziensis*	Malawi	♀+♂	4	VP2 (segment 2) VP3 (segment 3) VP5 (segment 6) NS3 (segment 10)
Alongshan virus	1	F10	*O. erraticus*	Spain	♀	1	VP2-3 (segment 4)

We further identified 7 contigs (5.4%) of NUMV (*Chuviridae*), implicated in human febrile diseases associated with tick bites [36], in a field collected *I. persulcatus* transcriptome, encompassing various regions in the NUMV genome (Table 2). ASFV (*Asfarviridae*) and AHSV3 (*Sedoreoviridae*) were observed in a *R. zambeziensis* transcriptome, with various regions of the virus genomes represented in the contigs. ASFV was identified from a field collected soft tick *Ornithodoros moubata* transcriptome as well, with a total of 5 contigs (3.9%) in the dataset. Finally, a single contig of ALSV (*Flaviviridae*) genome segment 4 was generated in the transcriptome of another field collected soft tick *O. erraticus* in Spain (S2 Table).

## Discussion

In this proof-of-concept study, our findings demonstrate that publicly available tick transcriptome data can be leveraged as low-cost resource to monitor the geographical expansion and new tick species associations of tick-borne viruses. Overall, we generated 127 contigs of DNA and RNA virus genomes belonging to 15 families from 35 tick transcriptomes, where 32.8% of the contigs comprise known human and animal pathogens. BJNV was the most prevalent tick-borne pathogenic virus, identified in 19.5% of the virus contigs and in 22.8% of the transcriptomes. Classified in *Norwavirus beijiense* species (genus *Norwavirus*, family *Nairoviridae*), the BJNV genome comprises two segments, encoding for the viral nucleoprotein (S segment) and replicase (RNA-dependent RNA polymerase, L segment), and lacks the M segment present in other nairovirus genera [35]. It is described as the causative agent of tick-associated human febrile diseases occurring in the Inner Mongolia autonomous region of China [5]. In the region, high rates of virus exposure identified by virus-specific antibodies were documented in humans, sheep and cattle. Moreover, viral pathogenicity was observed in cell culture and experimental animal inoculations. Screening of ticks from different regions of China revealed BJNV genomes in many *Dermacentor*, *Haemaphysalis*, and *Ixodes* spp. (including *I. persulcatus*), and most recently, in *Rhipicephalus sanguineus* sensu lato [5,33,37,38]. We detected BJNV in colony-reared *I. ricinus* from Switzerland and field collected *I. persulcatus* from China, encompassing various developmental stages, feeding status and sex. BJNV has not been previously reported from Europe or in colony-reared ticks. Maximum likelihood analyses using longer contigs of viral genome segments provided further evidence for virus identification in field collected samples, while findings in colony samples might indicate co-infections of *Norwavirus beijiense* and *Norwavirus grotenhoutense* species in the *Norwavirus* genus. Currently, *Norwavirus grotenhoutense* species includes only Grotenhout virus, described in *I. ricinus* ticks from Belgium [39], and closely related viruses such as Pustyn virus and Norway nairovirus 1 documented in Bulgaria, Poland and Norway [7,40]. In any case, these findings warrant further screening for this virus species accommodating a documented pathogen in Europe.

We further detected NUMV, comprising 5.4% of the total contigs, in a field collected *I. persulcatus* transcriptome (Tables 1 and S2). NUMV is the only chuvirus with potential medical significance demonstrated so far [36]. Chuviruses (family *Chuviridae*) are single-stranded negative sense RNA viruses, with diverse genome topologies including unsegmented, segmented, linear, or circular genomes [41,42]. Widespread around the globe, they are discovered by MS and subsequently identified in arachnids including ticks and spiders as well as several other insects, barnacles, decapod crustaceans and reptiles. The index case had presented non-specific febrile disease following tick bites and the resulting investigations documented 54 patients with detectable virus genomes and antibody responses during 2017–2019 [36]. Therefore, NUMV is a strong candidate to be included in the list of recently described viruses to be considered an etiological agent in the workup of cases with unknown etiology. In China, it is found in many *Ixodes* and *Haemaphysalis* species and isolated from *I. persulcatus* [36], in parallel with our transcriptome findings.

Analysis of the transcriptome data revealed ASFV, comprising of the 3.9% contigs and 5.7% of the datasets (Tables 2 and S2). ASFV produces a tick-transmitted hemorrhagic fever with up to 100% mortality rate in domestic and feral swine, resulting in tremendous socioeconomic impact [43]. It is endemic in Africa affecting many countries and has emerged in Europe, Asia, and in the Americas [44]. Soft ticks of the genus *Ornithodoros* are the only recognized ASFV biological vectors and *I. ricinus* and *D. reticulatus* are unlikely to be relevant for transmission [45]. Our finding of ASFV in the field collected *O. moubata* transcriptome coincides with virus epidemiology, although detection in a *R. zambeziensis* colony is perplexing. It remains to be described whether this species has potential to contribute to virus circulation or merely represents a coincidental finding. Another unexpected pathogen, AHSV-3, was also present in the same transcriptome data, with four of ten virus genome segments being represented as contigs (Table 2). African horse sickness is widely distributed in sub-Saharan Africa and affects several equids, with detrimental impact across working equids and domestic horse industries [46]. The primary vectors responsible for transmission are the *Culicoides* biting midges, with evidence for involvement of mosquitoes and/or ticks [47]. Hence, the impact of virus detection in the colony requires further assessment for local AHSV-3 epidemiology and control.

We identified another tick-borne pathogen, ALSV, represented as a single contig of genome segment 4, encoding for the virus structural proteins VP2/3 in the study dataset [48]. ALSV is classified in the Jingmenvirus group of the family *Flaviviridae* and possesses a positive-sense single-stranded RNA genome in four segments. Human infections are described as tick bite-associated, non-specific febrile disease [3]. ALSV has been reported in *Ixodes*, *Dermacentor* and *Haemaphysalis* ticks from many Eurasian countries such China, Finland, France, Germany, Russia, Serbia and Switzerland, with evidence of exposure in sheep, cattle, and deer [3,49–52]. Moreover, vector competence of *I. ricinus* and *Dermacentor reticulatus* could be experimentally demonstrated [52]. However, ALSV has not been so far reported in Argasid ticks of the *O. erraticus* complex, nor from Spain. Capable of transmitting several important livestock and human pathogens, including ASFV and *Borrelia hispanica*, the *O. erraticus* tick complex is reported from the Iberian Peninsula, North and West Africa, and western Asia [53]. A possible genome integration should also be considered, given that Jingmen tick virus, a tick-borne human pathogen closely-related to ALSV, was previously reported to partially integrate into *I. ricinus* genome [54]. Further tick screening is needed to elucidate presence and probable replication of ALSV in *O. erraticus* and possible impact on human/animal health.

Our exploration further revealed other viruses not directly associated with tick lifecycle in nature (Table 1), such as Triatoma virus (*Dicistroviridae*) that has so far only been described in wild and colony populations of *Triatominae* (kissing bugs) [55]. The detection of this virus in field collected *O. rostratus* possibly resulted from shared host feeding in the environment. Similarly, presence of *Citrus tristeza* virus, the agent of the economically damaging plant disease of the *Citrus* genus and vectored by certain aphid species, in colony-reared *R. zambeziensis* suggests a potential environmental origin. Tick cell line ISE6 transcriptomes further revealed bunyaviruses and iflaviruses, previously reported in identical cell lines, with scarce information about modes of infection, persistence, and impact on tick cells or individual ticks [27]. Interestingly, we observed particular tick cell lines to generate densovirus contigs, isolated from many mosquito species of the genera *Culex* and *Aedes* as well as mosquito cell lines [56,57]. These viruses have not been reported in ticks or tick cell lines previously and their origins remain obscure.

In this study, we used a straightforward strategy involving *de novo* assembly, followed by aligning to the NCBI non-redundant protein database and taxonomical binning by robust tools, for generating virus contigs in tick transcriptomes selected for analysis [58]. Comparable approaches have been used on mosquito, mammalian and avian transcriptomes, mostly with the prime objective of novel virus discovery [13,59]. Other strategies include querying well-conserved amino acid imprints in RNA-dependent RNA polymerase enzymes, a hallmark gene of RNA viruses that lack a DNA stage during replication [60]. Screening publicly available data by aligning to a custom curated database with tick-borne virus reference genomes of interest was performed as well, with a subsequent risk prediction for zoonotic infections [61]. Currently, there is no standardized or optimized approach or toolset for transcriptome/metagenome data processing for broad range pathogen screening or virus discovery. When previously generated data is recycled for other purposes, the quality of the initial sequencing, frequently intended for targets other than viruses become an important bottleneck and may result in lower virus abundance and shorter contig assemblies [62]. Complete virus genome assemblies are often difficult to generate, requiring scaffold assemblies and contig extensions to maximize coverage. Moreover, such assemblies carry the risk of being derived from multiple virus populations, leading to artificially generated chimeric genomes, which can occur between closely related exogenous virus populations and with integrated but sufficiently similar sequences in the host genomes [62]. Therefore, appropriate checks for data accuracy and integrity are essential, especially in novel virus discovery attempts. In this study, with aims to explore existing data to uncover tick-borne viral pathogens, we examined the generated virus contigs directly, utilizing commonly-used tools and straightforward approaches, for unbiased data interpretation and better reproducibility.

A significant bottleneck is the insufficient metadata associated with some publicly available transcriptomes, which can significantly hamper understanding the origin and nature of the sample. Most public repositories including NCBI do not currently impose stringent criteria on metadata to be provided during submissions. Also, not all information embedded in the submission is searchable, preventing robust selection of transcriptomes for further analysis. We experienced these issues during our initial selection, which resulted in our manual assessment of metadata and additional, non-standardized selection of entries to be included in the downstream process, a major limitation of the current study. Nevertheless, our study demonstrates that the data is suitable for pathogen screening and would be of significance with more stringent and reproducible data selection criteria. Moreover, it has advantages for identifying known tick-borne viruses, such as directly following NCBI database updates and generating contigs encompassing various regions of the virus genomes; therefore, multi-layered evidence for pathogen presence. Integrated or endogenized viral sequences in tick chromosomes have been documented for many viruses, which poses a challenge for data interpretation as well as nucleic acid assays commonly used in screening, and might require additional genome targets to confirm presence of replication competent viruses [54,63]. In any case, the findings of *in silico* explorations such as in this study must be interpreted with caution and pathogen detections should preferably be confirmed by follow-up screening using field collected samples and standardized assays.

As genetic databases are estimated to double in almost every 18 months (https://www.ncbi.nlm.nih.gov/genbank/statistics/; accessed May 4 2023), re-use of original datasets might provide a low-cost option for monitoring the spread of global viruses, and help elucidate new insect vector and vertebrate host associations. Our findings demonstrate that previously generated transcriptome data can be leveraged for detecting tick-borne viruses, as exemplified by preliminary evidence for the several human pathogens. Monitoring tick-borne viruses using publicly available data can significantly augment surveillance efforts, highlighting regions of probable spillover and identifying targets for screening.

## Supporting information

S1 Appendix Sequences of virus contigs generated in the study. Sample information is provided in S1 Table.
(XLSX)

S1 Fig The maximum likelihood tree of the Norwavirus replicase (A: L segment, 540 amino acids), and nucleoprotein (B: S segment, 450 amino acids), constructed using Jones-Taylor-Thornton model with uniform rates for 500 replications. Sequences obtained in the study are marked and indicated with sample identifiers. Virus strains are indicated by GenBank accession number, name and isolate identifier. Crimean-Congo hemorrhagic fever virus isolate Matin was included as an outgroup.
(PDF)

S1 Table List of transcriptomes processed for virus contig generation. (XLSX)

S2 Table Sample information, virus contigs and top BLAST hits. Longer contigs produced by reference mapping are indicated as Lcontig. Contigs with insertion/deletions (InDel) were marked.
(XSLX)
