# Supplementary material for: Exploring the potential of tick transcriptomes for virus screening: A data reuse approach for tick-borne virus surveillance
Source: PLoS Negl Trop Dis. 2025 Mar 6;19(3):e0012907. doi: 10.1371/journal.pntd.0012907 (PMC11922208; doi:10.1371/journal.pntd.0012907)
Supplement: S1 Fig — Sequences obtained in the study are marked and indicated with sample identifiers. Virus strains are indicated by GenBank accession number, name and isolate identifier. Crimean-Congo hemorrhagic fever virus isolate Matin was included as an outgroup. (PDF) [file pntd.0012907.s002.pdf]

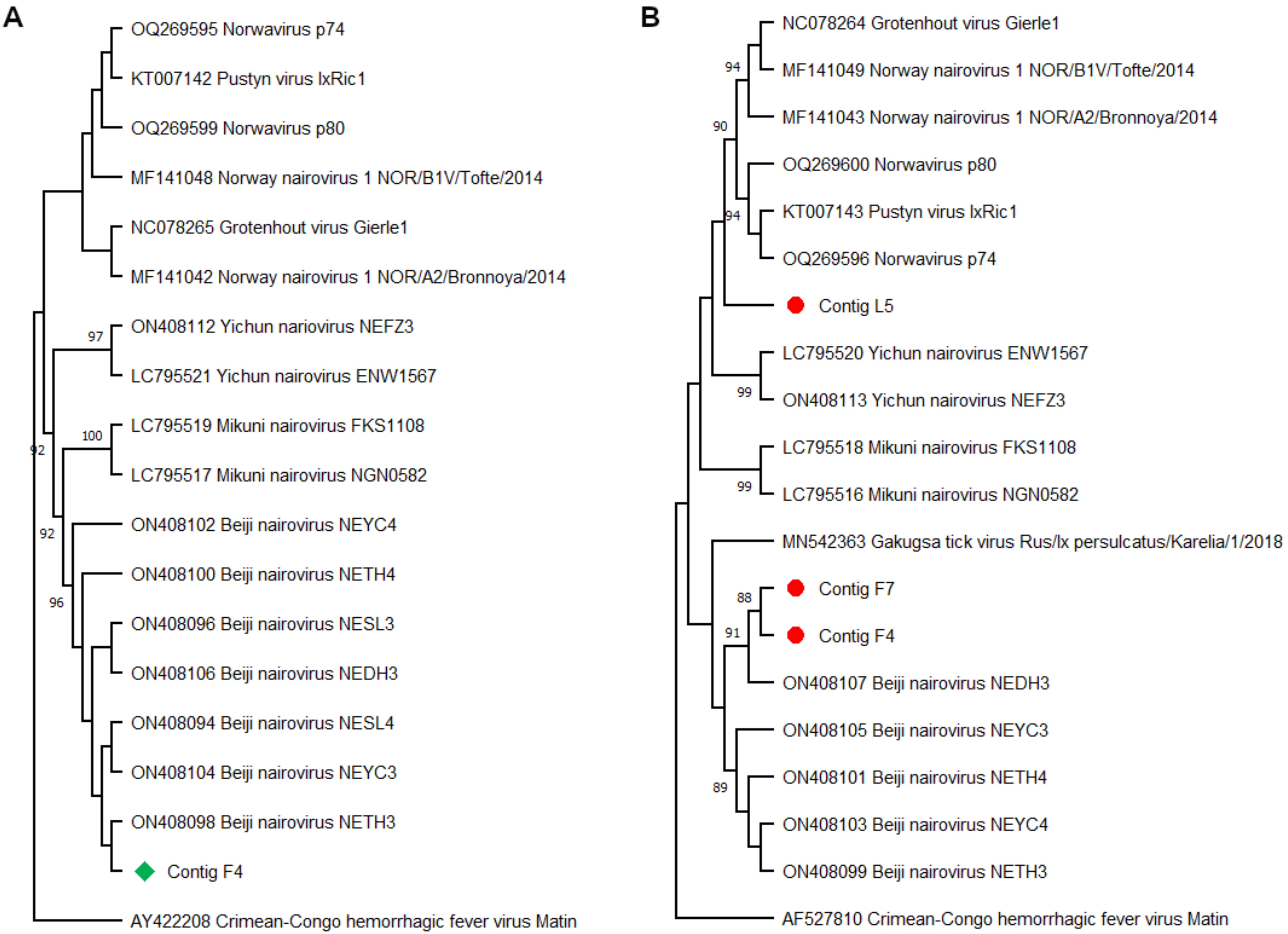

Figure S1: The maximum likelihood tree of the Norwavirus replicase (A: L segment, 540 amino acids), and nucleoprotein (B: S segment, 450 amino acids), constructed using Jones-Taylor-Thornton model with uniform rates for 500 replications. Sequences obtained in the study are marked and indicated with sample identifiers. Virus strains are indicated by GenBank accession number, name and isolate identifier. Crimean-Congo hemorrhagic fever virus isolate Matin was included as an outgroup.
